# Micro-CT and Histomorphometric Analysis of Degradability and New Bone Formation of Anodized Mg-Ca System

**DOI:** 10.3390/biomimetics10090583

**Published:** 2025-09-03

**Authors:** Jihyun Kim, Yoona Jung, Yong-Seok Lee, Seong-Won Choi, Geelsu Hwang, Kwidug Yun

**Affiliations:** 1 Department of Prosthodontics, School of Dentistry, Chonnam National University, Gwangju 61186, Republic of Korea; zh5542@hanmail.net (J.K.); yoonajung24@gmail.com (Y.J.); 2 Department of Mechanical Engineering, Konkuk University, Seoul 05029, Republic of Korea; yslee@konkuk.ac.kr; 3 Industry Support Center for Convergence Medical Devices, Chonnam National University Hospital, Gwangju 61469, Republic of Korea; magma1534@gmail.com; 4 Department of Preventive and Restorative Sciences, School of Dental Medicine, University of Pennsylvania, Philadelphia, PA 19104, USA; 5 Center for Innovation & Precision Dentistry, School of Dental Medicine, School of Engineering and Applied Sciences, University of Pennsylvania, Philadelphia, PA 19104, USA

**Keywords:** Mg alloy, anodic oxidation, surface treatment, degradability, degradable Mg alloys

## Abstract

The surface treatments and various magnesium alloys are applied to improve the fast degradation rate and resulting negative effects of magnesium alloys. This study aimed to assess the effect of anodic oxidation treatment of magnesium–calcium (Mg-Ca) systems by creating artificial bone defects in the tibia of rats. The cylinder magnesium implants were fabricated using a Mg-xCa (x = 0, 1, 5 wt.%) binary alloy. Degradability and new bone formation were observed at two and six weeks using micro-CT. Histomorphometric parameters were evaluated with Goldner’s trichrome staining. The degradation rate decreased depending on the amount of calcium added. The parameters related to bone formation revealed an increasing pattern depending on the addition of calcium, anodic oxidation, and time. The amount of absorbed magnesium to assess degradability of magnesium implants by the histomorphometric analysis revealed a high value in the untreated group at two and six weeks. Bone healing parameters increased depending on the amount of calcium added, anodic oxidation treatment, and region of interest (ROI—0.5 mm, 1.00 mm, 1.5 mm, and 2.0 mm). Biodegradable magnesium systems have the potential to replace bone screws and plates. Combination with calcium combined with anodization surface treatment can improve initial corrosion resistance and promote bone formation.

## 1. Introduction

Metal-based biomaterials mainly used for maxillofacial correction/reconstruction and fracture surgery have a semi-permanent advantage due to their high inactivity [1]. However, their long-term presence in the body may interfere with the growth of the cranial fascia in growing patients, and due to excessive robustness, the bone may be bent or pain and chronic inflammation may occur. Since it is difficult to obtain accurate information due to image distortion during postoperative imaging tests, materials that can be absorbed in vivo are being developed. This approach also addressed the disadvantage of requiring follow-up removal surgery after bone healing [2].

Mg-Ca systems directly align with the principles of biomimetics, which focuses on designing materials that imitate natural biological processes for regenerative purposes. Magnesium systems are inherently suited for this approach due to their biodegradable nature and the gradual release of bone-friendly ions (Mg and Ca) [3,4], allowing them to serve as a transient scaffold that degrades in vivo while being progressively replaced by new bone. This process fundamentally mimics the natural dynamic of bone remodeling. The study’s detailed investigation into the degradability and subsequent new bone formation on an anodized Mg-Ca surface further exemplifies a biomimetic approach, aiming to create an optimized microenvironment that actively promotes cellular activity and guides the body’s intrinsic healing mechanisms [4]. Thus, it contributes to the development of next-generation biomaterials for effective bone tissue regeneration.

Recent research has focused on developing biodegradable highly reactive–active metals with sufficient strength [5,6]. Among the biodegradable metals, magnesium (Mg) being studied is an inorganic component that constitutes the human body and is found in bone tissue in the living body without exhibiting inflammatory reaction or toxicity in the living body [7,8]. Moreover, it has advantages, including high biodegradability, lightweight, and excellent processability. The elastic modulus of Mg is significantly lower than that of other metals and most similar to bone, which can minimize the stress shielding phenomenon, one of the common causes of implant failure [9]. However, pure magnesium has low mechanical strength, releases high concentrations of hydrogen (H_2_) upon degradation [9], and rapidly corrodes in aqueous solutions containing chlorine ions (Cl-) in the human body [10,11]. The decrease in strength due to corrosion rapidly reduces the support required for the period of time required for magnesium implants to perform their functions in vivo. In addition, the bonding force between the bone and the implant is weakened, and may not support sufficient bone healing [12].

Studies have focused on improving the corrosion resistance and strength of metals by controlling the degradation rate in vivo with alloying metals [13], particularly Mg-Ca-based biodegradable systems, which are an essential element and nontoxic to human cells [13]. Ca is the main component of bone and has little toxicity. Calcium ions (Ca^2+^) generated during degradation can contribute to osteosynthesis, improve mechanical properties, and control corrosion rates when alloyed [14,15,16].

In addition anodization method that forms an oxide layer and a porous surface to form an oxide film is emerging as a method for controlling the rapid degradation of magnesium. The film increases bone-implant contact and facilitates osseointegration. Moreover, it can be used as a medium to transfer factors involved in bone regeneration into the bone [17]. A research report shows that bone healing is promoted by increasing osteoblast adhesion and activity through changes in the surface structure [18].

Some studies have reported improvements in the mechanical properties and biological analysis of anodized Mg-Ca systems [19]. However, few micro-CT and histomorphometric studies have assessed the degradation behavior and bone response of Mg systems placed into bone defects in experimental animals. This study aimed to evaluate the degradation and bone formation performance of magnesium implants by placing anodized Mg-Ca systems into the tibia of rats.

## 2. Materials and Methods

An Mg-xCa (x = 0, 1, 5 wt%) binary system was manufactured using an alloying element of calcium (99.9% Ca, Junsei Chemical Co., Ltd., Tokyo, Japan) and magnesium (99.9% Mg, SinoZheng Industrial Inc., Zhengzhou, China). The Mg-xCa binary system was manufactured in a vacuum arc melting furnace (Vacuum Arc Remelting Furnace, Ace Vacuum Co., Gunpo-si, Republic of Korea) between 700 and 810 °C in a 5.0 × 10^−5^ Torr vacuum. The molten metal with different components was poured into a steel frame preheated at 200 °C for 2 h. An alloy rod with a diameter of 15 mm was cut into a 2 mm diameter and 5 mm length cylindrical magnesium implant using a linear precision cutter (ISOMET^®^ 5000, Buehler, Lake Bluff, IL, USA). The specimens were cleaned and sterilized with ethylene oxide gas.

The experimental group was divided according to the surface treatment with anodization and classified based on the calcium content. Three samples for each group were prepared for observation after 2 and 6 weeks (36 samples) (Table 1).

Electrolyte concentration and treatment conditions used in the surface coating process of acid treatment and anodization were performed (Table 2). Anodization was performed by connecting magnesium to the anode and a platinum plate (3 mm × 4 mm × 0.1 mm) to the cathode (DC Power Supply VUPOWER-AK6003, ICAN, Korea). Calcium glucose (4 g/L), sodium hexamethaphosphate (3 g/L), and sodium hydroxide (6 g/L) were used as electrolytes (20–25 °C). Anodization was performed for 15 min by fixing the voltage to 120 V. The samples were washed with running water for 20 min and dried.

This study was conducted with the approval of the Chonnam National University Animal Experiment Ethics Committee (CNU LACUC-YB-2015-61). Male, 8-month-old Sprague-Dawley rats (*n* = 18, average body weight = 250 g) were used. After general anesthesia, the surgical site was disinfected with a 10% povidone-iodine solution. After exposing the tibia, a circular bone defect with a diameter of 2 mm was prepared. The specimens were placed, and the wound was sutured (Vicryl, Ethicon, Raritan, NJ, USA). X-ray imaging confirmed the placement location of the Mg implant. The rats were euthanized with carbon dioxide (CO_2_) at the 2nd and 6th weeks.

The implantation location of the specimen on the day of surgery was confirmed using radiological analysis, and the animals were sacrificed at 2 and 6 weeks after the surgery to evaluate the bone condition and occurrence of inflammation. A digital dental X-ray device (ELITYS X-ray system, Kodak, Lyon, France) was used. Radiographs were taken in the distal and medial directions of the tibia.

Parameter of micro-CT (SkyScan 1172, SkyScan, Kontich, Belgium) was shown in Table 3. Table 4 shows the degradation of the Mg implant and microarchitecture analysis of bone formation. To calculate the bone surface area, bone volume, and bone density, the region of interest (ROI) was designated as 0.5 mm, 1.0 mm, 1.5 mm, and 2.0 mm from the outermost point of the specimen surface.

The surface area of the absorbed cylindrical magnesium implant was calculated by subtracting the surface area of the remaining magnesium implant at 2 and 6 weeks from the surface area of the initial magnesium implant. The volume of the absorbed cylindrical magnesium implant (MV) was obtained by subtracting the volume of the initial magnesium implant measured at weeks 2 and 6 from the volume of the magnesium implant as a change in volume over time. The degradation rate of cylindrical magnesium implants (DR) was derived using the following equation, where ∆Vi is the volume change and Si is the surface area. ∆Xi, during the time interval, represents the degradation rate by the degradation width (i is the moment of observation).

$$DRi=\frac{∆Xi}{∆t},\;∆Xi=\frac{∆Vi\;}{Si}$$

(DRi: Degradation rate, ∆Xi: Decomposition width, ∆t: Time change, ∆Vi: Volume change, Si: Surface area)

Bone surface (BS), bone volume (BV), bone surface ratio (BS/BV), bone mineral density (BMD), trabecular thickness (Tb.Th), and trabecular number (Tb.N) were measured using a CT Analyzer Image analysis software (CT-An v1.16, SkyScan, Belgium).

Bone surface, the amount of bone generated between the implant and the surrounding bone, represents the quantitative measurements of bone formed between the surface of the cylindrical magnesium implant and the surrounding bone. Bone volume measured the volume of bone within the ROI range (0.5 mm, 1.0 mm, 1.5 mm, 2.00 mm), and the ratio of bone surface area to bone volume was quantified as the ratio of surface area of the volume. Bone mineral density was calculated by obtaining the Hounsfield unit (HU) values for the standard phantoms of 0.25 and 0.75 g/cm^2^ and calculating the HU value of the specimen to be measured by applying the equation.

$$BMD=\frac{HU-0.00935}{0.0442}g/㎠,\begin{array}{ccc} . & Min & Max\\ BMD & 0.25 & 0.75\\ HU & 0.32502 & 0.99150 \end{array}$$

The shape of the bone defect was analyzed with a three-dimensional stereoscopic image using CT Volume image analysis software (CT-Vol v2.3, SkyScan, Belgium) and DataViewer analysis software (DataViewer v1.5, SkyScan, Belgium).

After setting the ROI by dividing the cortical bone and the trabecular bone from the reconstructed two-dimensional image, the trabecular thickness and the trabecular number were quantitatively analyzed. The trabecular thickness, determined as the average thickness of trabecular bone structure, indicates quantification of the degree of bone filling. The trabecular number was measured as the number of strands fitting within a unit length in cross-section.

To analyze the degradability of cylindrical magnesium implants, a digital image was obtained using Panoramic 250 Flash III (3D Histech, Budapest, Hungary), and the image was acquired using CaseViewer software (ver. 2.0, 3D Histech, Hungary). The length of the contact area between the bone and implant was measured using Image J software 1.54p and Image Pro Plus 7.0.1 (Media Cybernetics Inc., Silver Springs, MD, USA) to analyze the healing pattern and degree of bone remodeling and calculate the bone-to-implant contact ratio and bone amount.

Each extracted tissue sample was dehydrated, penetrated with an ethanol and resin mixture (Technovit 7200, Heraeus Kulzer, Hanau, Germany), fixed, and embedded. The UV-cured specimen was attached to the slide using a diamond-cutting system (EXAKT 300 CP, Norderstedt, Germany). The final step was the Goldner trimming (Goldner’s trichrome stain) for histomorphometric observation of the bone healing pattern and the degree of remodeling.

After measuring the area of whole cylindrical magnesium implants, the amount of residual magnesium implant was subtracted from the total magnesium implant area and expressed numerically to quantify the result. The substituted equation is as follows.

$$Amount\;of\;absorbed\;magnesium=\frac{Amount\;of\;total\;magnesium-Amount\;of\;residual\;magnesium}{Amount\;of\;total\;magnesium}$$

The BIC value was measured using an optical microscope with 100× magnification. After measuring the surface length of the total magnesium cylinder, the value obtained by subtracting the length of the total surface area without bone contact from the overall length of the Mg implant was expressed as a percentage.

$$BIC(bone\;to\;implant\;contact\;ratio,\%)=\frac{Length\;on\;contacted\;cylinder\;implant\;side}{Overall\;length\;on\;cylinder\;implant\;side}\times 100$$

The BA value between the bone and the implant was obtained at 100× magnification, and the total area was measured by selecting the part of the implant surface where bone contact occurred continuously and drawing an outline between the lines according to the ROI setting range (0.5 mm, 1.0 mm, 1.5 mm, and 2.0 mm from the outer point of the surface). The bone formation area was measured, and the ratio was calculated to obtain the bone amount between the surrounding bone and the implant.

$$BA\left(bone\;area,\%\right)=\frac{Bone\;area\;of\;according\;to\;ROI}{Total\;area\;of\;according\;to\;ROI}\times 100$$

A nonparametric statistical test was performed using IBM SPSS Statistical 23 software (SPSS Version 23, SPSS Inc., Chicago, IL, USA). A Friedman test determined the differences for each group according to the amount of calcium added, anodization treatment, time, and ROI settings. A post-test was performed using the Wilcoxon signed-rank test. *p* < 0.05 was set as the significant level among groups.

## 3. Results

**Figure 1:**
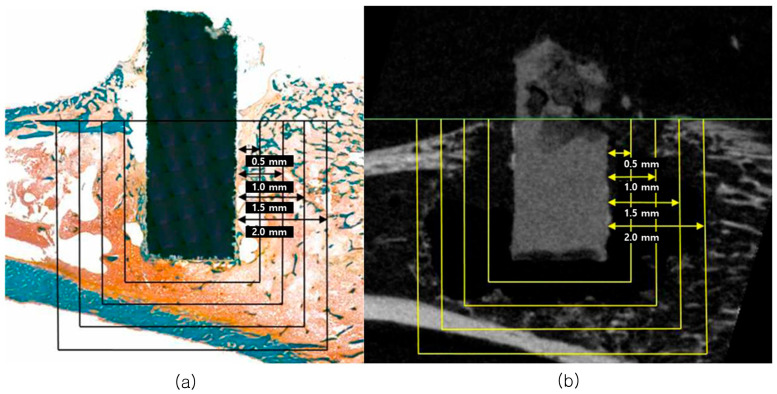
Bone amount of tissue section (**a**) and radiographic image (**b**) based on ROI range at 0.5 mm, 1.0 mm, 1.5 mm, and 2.0 mm apart from the innermost point of the cylinder magnesium implant surface.

Experiment results were presented in two ways: micro-CT analysis and histomorphometric analysis according to the compositions of the control and experimental groups. The parameters, according to bone, were calculated using the sections of Mg implant-surrounding tissues called ROI setting range 0.5 mm, 1.0 mm, 1.5 mm, and 2.0 mm (Figure 1).

### 3.1. Clinical Evaluation

Macroscopic findings showed that hydrogen bubbles were formed in two out of three cylindrical magnesium implants in the MN group and one out of three cylindrical magnesium implants in the MA group at two weeks, causing the defects to swell considerably; the detachment of the implanted cylindrical magnesium implants was also confirmed. However, in all groups except this one, the wounds healed well without any dehiscence, infection, or fracture at two and six weeks. The cylindrical magnesium implants were confirmed to heal well within the bone without external exposure or specific inflammatory findings.

**Figure 2:**
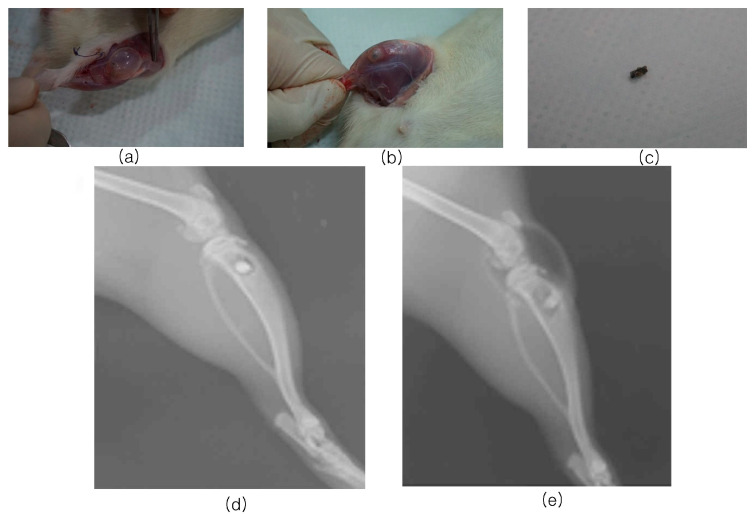
Digital photograph of MN and MA groups at two weeks. (**a**) MN group tibia area with bubble generation, (**b**) MA group tibia area with bubble generation, (**c**) cylindrical magnesium implants missing due to bubbles, radiographs of two weeks post-surgery. (**d**) 5CMA, normal rat, (**e**) MA, bubbled rat.

X-ray imaging revealed that two out of three cylindrical magnesium implants in the MN group and one of three cylindrical magnesium implants in the MA group had hydrogen bubbles (Figure 2). Hydrogen bubbles occurred in the first two weeks after implantation, but no hydrogen bubbles occurred at six weeks, indicating no effect in the later period after implantation. Implants were placed in appropriate positions without inflammation or hydrogen bubbles in all other groups.

### 3.2. Micro-CT Analysis

#### 3.2.1. Mg Implant Analysis

**Figure 3:**
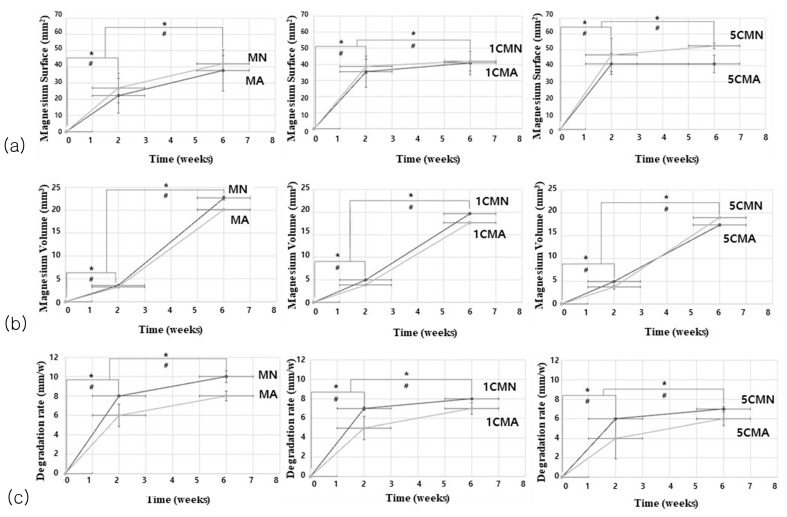
In vivo degradation performance of magnesium implant cylinder during the period, assessed via μCT measurements: (**a**) absorbed magnesium surface, (**b**) absorbed magnesium volume, (**c**) magnesium implant cylinder degradation rate. [*: There was a significant difference between groups according to time, #: there was a significant difference between groups according to anodic oxidation treatment].

The surface area (MS) of a Mg implant that degraded and absorbed over time was calculated (Figure 3a). Although an increased calcium amount upped the absorbed surface area in Mg implants, there was no significant difference (*p* > 0.05). Among all groups receiving the anodizing surface treatment with different amounts of calcium, the surface area of the absorbed implants in the MN groups was significantly higher than that in the MA groups and increased at 6 weeks (the later stage of degradation) compared to two weeks (the early stage of degradation). The absorbed surface area values according to time and anodizing surface treatment were statistically significant at *p* < 0.05.

This study evaluated the total volume of the magnesium implant (MV) degraded and absorbed over time (Figure 3b). In the second week, the MN group in all graphs had higher absorbed implant volume values. However, in the 6th week, the 5CMA group exhibited significantly higher values than the 5CMN group. Overall, the volume of implants absorbed at six weeks was greater than at two weeks, with a statistically significant difference (*p* < 0.05). As the amount of added calcium increased, the volume of absorbed magnesium implant decreased, but this difference was statistically insignificant (*p* > 0.05).

The degradation rate of cylindrical magnesium implant (DR) value of Mg implants was obtained based on anodization surface treatment (Figure 3c). Increased calcium was associated with a low degradation rate; however, there was no significant difference (*p* > 0.05). The MN group showed a significantly higher degradation rate than the MA group (*p* < 0.05). Implant degradation increased rapidly in the first two weeks; however, the degradation rate tended to increase gradually up to six weeks.

#### 3.2.2. Bone Formation Analysis

**Figure 4:**
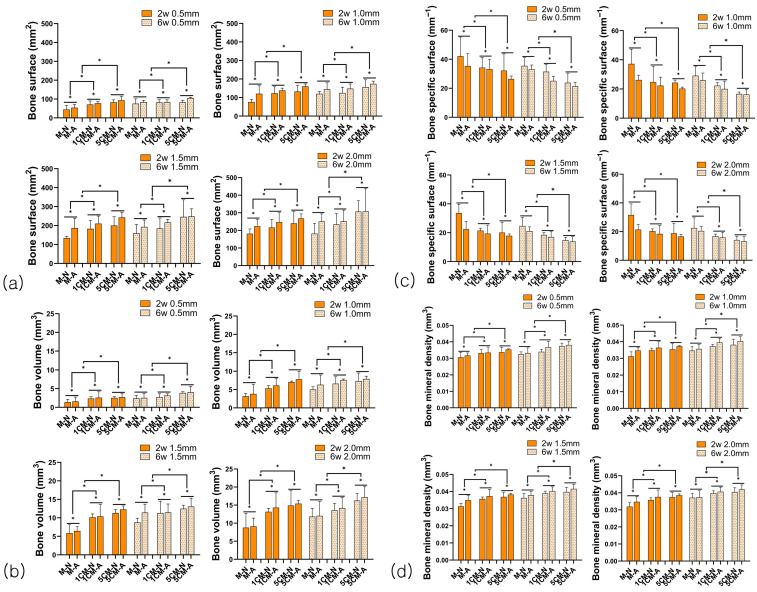
Bone surface (**a**), bone volume (**b**), bone specific surface (**c**), bone mineral density (**d**) at each region of interest. [*: there was significant difference between each group at any region of interest].

The bone surface area (BS) values within the ROI range tended to increase as the amount of incorporated calcium increased (Figure 4a). All BS measurements showed a significant increase in the MA group compared to the MN group, and the 5CMA group had the highest BS value. The BS values in both the MN and MA groups showed a significant increase in the sixth week compared to the second week. The numerical value significantly improved as the range expanded based on the ROI setting criteria, with statistically significant differences depending on the amount of calcium added, anodized surface treatment, time, and ROI range (*p* < 0.05).

As the amount of calcium added increased, the bone volume (BV) values tended to increase, and the bone volume values were significantly higher in the MA group than in the MN group (Figure 4b). The values were higher in the six weeks after degradation than in the two weeks after degradation. The values significantly increased as the ROI range widened, with statistically significant differences depending on the amount of calcium added, anodized surface treatment, time, and ROI range (*p* < 0.05).

The significant decrease in the ratio of bone surface area to bone volume (BS/BV) value was confirmed depending on the amount of calcium added (Figure 4c). The value decreased at six weeks after degradation compared to the initial two weeks. In particular, the 5CM group at two and six weeks showed a higher decrease than the MN group. The MA group showed lower values than the MN group in all graphs. A wider ROI range was associated with a more significant decrease (*p* < 0.05).

The bone mineral density (BMD) value within the ROI range increased as calcium was added to the alloy (Figure 4d). The BMD value of the MA group significantly increased in all graphs. Degradation measurements in 6 weeks increased in all groups compared to degradation in two weeks. The BMD values significantly increased as the ROI range widened. There was a significant difference depending on the amount of calcium added, anodization surface treatment, time, and ROI range (*p* < 0.05).

**Figure 5:**
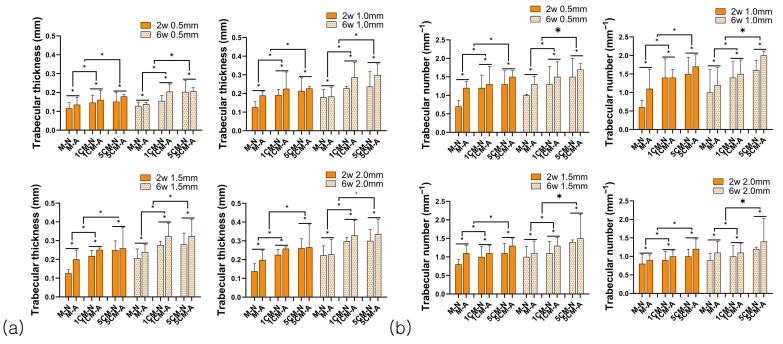
Trabecular thickness (**a**) and trabecular number (**b**) at each region of interest. [*: there was significant difference between each group at any region of interest].

The graph shows the trabecular bone thickness (Tb.Th), quantified as a distribution of average thickness and thickness for each section (Figure 5a). As the amount of calcium added increased, the Tb.Th value also increased. The MA group had a higher value for trabecular bone thickness than the MN group, and the value significantly improved at six weeks of degradation rather than two weeks (early stage of degradation). The ROI value significantly increased as the range widened, with a significant difference according to the amount of calcium added, anodization surface treatment, time, and ROI range (*p* < 0.05).

The graph numerically represents the number of strands that fit into a specific unit length (Figure 5b). As the calcium content increased, the number of trabecular bone (Tb.N) increased, and the value significantly improved in the MA group compared to the MN group. It showed a significant increase at six weeks compared to two weeks, and it was expected that the arrangement would become more solid in the process of bone remodeling than in the initial fixation. Moreover, the value of the number of bone trabeculae significantly increased as the ROI range widened, with a statistically significant difference depending on the amount of calcium added, anodized surface treatment, time, and ROI range (*p* < 0.05).

### 3.3. Histomorphometric Analysis

#### 3.3.1. Goldner Trichrome Stain

**Figure 6:**
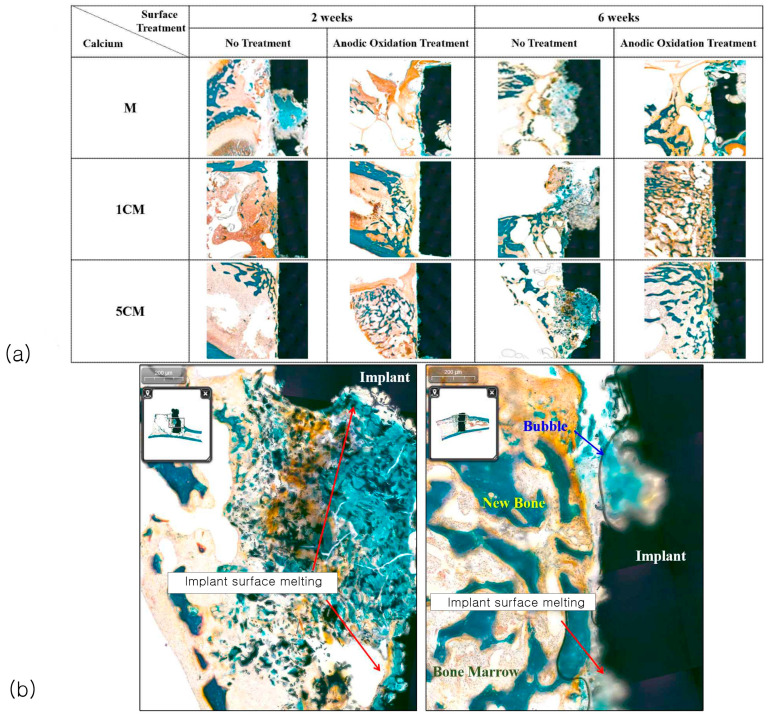
Histologic images of sectioned specimen (**a**), histologic images of treated and untreated specimens in six weeks (**b**) 5CMN-left and 5CMA-right are shown.

Figure 6a shows the results of histomorphometric analysis. Goldner’s trichrome stain was used to analyze the healing pattern and degree of bone remodeling. Inflammatory cells were scarce throughout the defect area, but some new bone and osteocytes were observed. Implant degradation was more active in the MN group than in the MA group, and it was confirmed that more was dissolved at six weeks compared to the two-week specimen after implantation. In particular, the MN group showed a high reactivity and degradation rate. However, as the amount of calcium added increased, the degradation rate decreased significantly, and the 5CM group was most similar to the appearance of the cylindrical magnesium implant before implantation compared to the implants in other groups.

Figure 6b shows an enlarged photograph of the lower part of the tibia in the 5CM group at six weeks. The 5CMA group showed dissolved surfaces and partially formed new bone around the implants with osteocyte migration. Some bubbles observed around the implant surface were identified as implant degradation products generated during tissue sectioning. However, the 5CMN group showed no osteocyte formation due to partial degradation of the magnesium implants.

#### 3.3.2. The Amount of Absorbed Cylindrical Mg Implant, Bone-Implant Contact Rate (BIC), and Bone Amount (BA)

**Figure 7:**
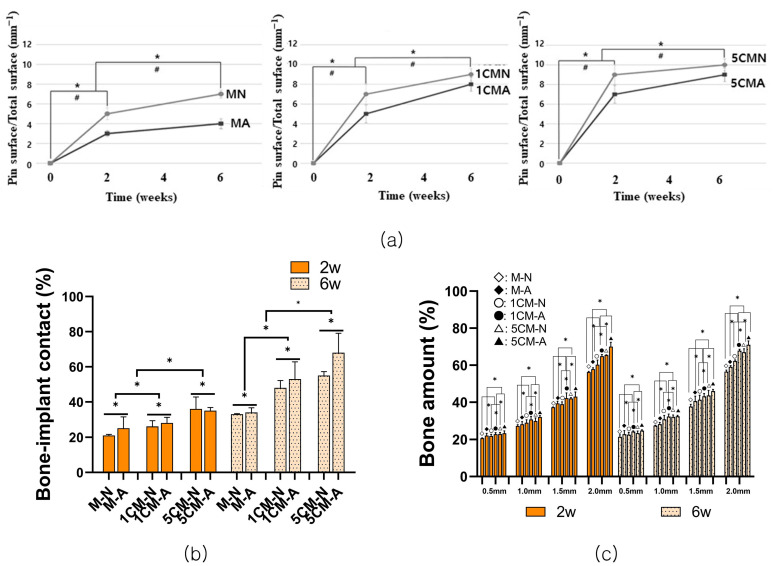
Graph showing the amount of absorbed alloy. (**a**) Diagram showing the percentages of implant surface contacted to bone; (**b**) diagram showing the percentages of bone formation area inside the cylinder magnesium implant; (**c**) [*: there was significant difference among groups according to time; #: there was significant difference between the each group according to anodic oxidation treatment.].

Figure 7a shows the absorbed Mg implant using the volume fraction of the alloy as a function of the amount of Mg absorbed. The amount of absorbed magnesium implant decreased with the anodization surface treatment, and the MA group showed a significantly higher amount of remaining implant than the MN group, particularly at six weeks than at two weeks. The 5CMN group showed a higher amount of absorbed Mg implant 6 weeks after degradation. As the amount of calcium added increased, the amount of absorbed magnesium implant increased but was statistically insignificant (*p* > 0.05).

The length of the bone-implant contact area increased at six weeks rather than two weeks, and the contact rate showed a significant increase with increased amounts of calcium in all graphs (Figure 7b). The MA group showed a higher value than the MN group, confirming that the contact rate between the bone and cylindrical magnesium implant improved by the anodizing surface treatment. The increase in the contact rate between the bone and the implant, according to the amount of calcium added, anodizing surface treatment, and time, showed a significant difference (*p* < 0.05).

As the ROI range widened, the bone amount (BA) increased, showing a significant increase with increased amounts of calcium (Figure 7c). The bone amount tended to increase significantly in the MA group compared to the MN group. The results showed a statistically significant difference according to the amount of calcium added, anodizing surface treatment, time, and ROI range (*p* < 0.05).

## 4. Discussion

Various methods have been explored to control the rapid degradation rate of magnesium. Several surface treatment methods control the degradation rate by increasing the thickness of the implant film layer. Surface treatment techniques, such as anodization, can improve corrosion resistance by creating a desirable surface area and controlling the initial degradation rate [20]. In particular, anodization has effectively increased implant surface area and modified the surface shape to promote osseointegration. This surface modification is consistent with the findings in this study, as the MN group showed a significantly higher degradation rate than the MA group.

Anodization is a surface treatment method that uses an Mg implant as the anode and platinum as the cathode to produce an oxide film on the implant surface through galvanic action by passing an electric current in an electrolyte solution [20]. The anodized surface layer obtained through this electrochemical process is crucial in bone healing as it improves biocompatibility by inhibiting metal dissolution in the body [21]. Moreover, it has shown rapid bone healing and increased implant stability in animal experiments due to the porous surface structure [22].

Although previous research has evaluated osseointegration using surface-treated implants in various ways [18], there are few micro-CT and histometric studies on alloying and anodization surface treatments. Therefore, this study aimed to analyze the degradability and degree of new bone formation of magnesium implants by coating the surface with anodization treatment on Mg-Ca system implants.

Magnesium-based bone implants react with water, releasing hydrogen gas (H_2_), and degrade by forming magnesium oxide. Magnesium oxide reacts with water to form magnesium hydroxide [23,24]. Increased levels of magnesium hydroxide in the body raise the surrounding pH, creating an alkaline environment known to enhance bone response [25]. Furthermore, magnesium hydroxide demonstrates good biocompatibility and osteoconductive properties [26].

Radiological analysis of the magnesium implants revealed hydrogen gas (H_2_) production in the pure magnesium group at the beginning of degradation at two weeks. However, hydrogen gas (H_2_) generation was not observed as the calcium content increased, even six weeks after degradation. Xue et al. reported bubble formation within one month of implantation [27], consistent with the results of this study. Hydrogen gas diffuses through the tissues and is eliminated in the body, such as in the bloodstream and kidneys [28,29]. Thus, the initial degradation rate of magnesium at two weeks was too fast, and hydrogen gas continued to be generated before being absorbed and disappearing in the body. At the end of six weeks, the degradation rate slowed, and hydrogen gas was naturally absorbed into the body. Magnesium implants degrade quickly in aqueous solutions, particularly before a stable degradation layer forms. The accumulation of corrosion products, such as magnesium hydroxide and calcium phosphate, forms a partially protective surface layer that slows down further degradation. However, this layer is often porous and may gradually break down or dissolve in vivo, allowing degradation to continue at a slower but still progressive rate up to six weeks. Additionally, bubbles were generated in 3 of 36 cylindrical magnesium implants on radiographic images. Other magnesium implants healed without wounds, infection, and inflammation during the observation period.

The degradability of magnesium implants evaluated by micro-CT analysis showed that the surface area and volume of the absorbed magnesium implant significantly decreased on anodized surfaces. In the graph showing the volume of absorbed magnesium implants, the 5CMA group showed a significantly higher value than the 5CMN group at 6 weeks, which was the later stage of degradation. The rapid increase in magnesium degradation can be explained by pitting corrosion [30]. Some alloys can rapidly degrade due to localized corrosion damage, and localized coating destruction accelerates corrosion [31]. Localized damage is a well-known phenomenon in untreated magnesium alloys [32], but little is known about the stable resistance to localized corrosion in body fluids of the group treated with micro-arc oxidation (MAO). Therefore, additional research on the rapid degrading mechanism caused by surface treatment is necessary.

In this study, the degradation rate of Mg implants was significantly reduced in all groups after two weeks of initial degradation and six weeks of final degradation after anodization. Gu et al. showed that micro-arc oxidation surface treatment (based on plasma electrolyte oxidation technology) can reduce the corrosion rate by up to 90% [33]. Fischerauer et al. found that volume loss and degradation rate decreased when the surface was treated with micro-arc oxidation [34]. The average degradation rate of the micro-arc oxidation surface treatment group at the initial stage after implantation was 0.25 mm/a, and that of the untreated group was 1.7 mm/a [34]. Chen et al. noted an average degradation rate of 1 mm/a for eight weeks, while the composite-coated group showed a degradation rate of only 0.13 mm/a [30]. Thus, the effect of the micro-arc oxidation coating was clearly demonstrated by rapid degradation in the untreated group. The decomposition rate in the untreated group of this study was more rapid than that in the anodized group in the first two weeks of degradation because anodization produced a protective oxide layer on the surface, which controlled the rapid initial degradation rate of the alloy. The pH concentration increased due to the increase in magnesium hydroxide (Mg(OH)_2_) generated during magnesium implant degradation, and the in vivo environment reached a pH of seven or higher. Such an alkaline environment delayed further degradation because it promoted the growth of the protective implant oxide layer [35,36]. The dense oxide layer reduces the degradation rate after two weeks of initial corrosion and maintains a lower rate through six weeks, aided by the subsequent deposition of calcium phosphate minerals that further protect the surface. Material properties, such as degradation rate, can be controlled by processing, alloying, and coatings [29,37]. In particular, surface treatment can effectively increase the corrosion resistance of magnesium alloys during initial degradation and increase surface biological activity, improving the degradation rate later [34].

Micro-CT analysis shows that bone surface area and volume increase can improve stability during healing. Witte et al. reported that the degradable magnesium scaffold promoted bone formation, and more mature bone formed around the degradable magnesium scaffold [38]. They inferred that the corrosion rate slowed because the protective magnesium oxide layer and magnesium hydroxide surface layer formed after one week, and the bone volume increased later [39]. The results of this study confirmed that the bone surface area and volume significantly improved with surface anodization. Depending on the various methods of surface treatment, the components formed on the surface induce changes in surface energy and roughness [40]. Changes in surface roughness play an important role in enhanced surface cell attachment and the rate of osseointegration [41,42]. Thus, the rise in surface roughness with increasing Ca concentration can be attributed to the formation of precipitates during anodization in sodium hydroxide and sodium phosphate electrolytes, which induced cell adhesion and increased osseointegration [43,44]. This study showed that the surface area and volume of bone tended to increase with increased amounts of calcium and anodization treatment showed as higher bone volume in MA group compare to MN group. Calcium (Ca) is an essential and non-toxic mineral in the human body. Calcium ions (Ca^2+^) generated during decomposition can facilitate osseointegration [45,46]. Moreover, it can improve mechanical properties and corrosion rates by enabling the formation of Mg-Ca phosphates [39,42,47]. Our study results showed that calcium added to the magnesium systems promoted bone formation and influenced bone bonding.

Since the implant surface is exposed to the physiological environment, an initial thin layer of magnesium hydroxide is produced, which can be partially destroyed by chloride ions within the body. The controlled degradation of Mg–Ca systems leads to a sustained release of Mg^2+^ and Ca^2+^ ions, both of which play a significant role in bone regeneration. While magnesium ions enhance osteoblast proliferation and angiogenesis through signaling pathways such as Wnt/β-catenin and enhance alkaline phosphatase activity to enable early matrix mineralization, calcium ions contribute to osteoblast differentiation and hydroxyapatite crystal deposition via calcium-sensing receptor signaling [48,49]. The local increase in pH during degradation allows the deposition of a bone-like apatite layer on the implant surface, which improves cell adhesion and serves as a biologic interface for the growth of new bone [50,51]. The process supports the sequential phases of bone healing: early inflammation modulation, recruitment and proliferation of bone-forming cells, and ultimate maturation of mineralized tissue.

According to these mechanisms, findings in this study indicate the higher the value of the trabecular bone thickness, which shows the distribution of the thickness by section under micro-CT analysis, and the bone trabecular number, which quantifies the point where the cancellous bone strands overlap, the higher the bone density and bone reinforcement. Witte et al. reported that increased bone trabeculae around magnesium scaffolds formed mature bone during osseointegration and showed better mechanical support [38]. In this study, bone trabecular thickness and number increased with increased calcium content, and the values improved in the anodized group compared to the untreated group. This finding indicated that surface treatment improved bone density, increasing the overlapping points with bone and thereby enhancing the mechanical strength of bone. Moreover, bone trabecular thickness and number significantly improved at six weeks compared to two weeks after degradation. It is judged that the thickness and number of trabecular bone become more solid in their arrangement during the bone remodeling process than during the initial fixation period.

Micro-CT analysis showed that bone density significantly increased with calcium addition and anodization surface treatment. Since new bone is formed as the implant dissolves, the increase in bone density over time can be confirmed by the mineralization of new tissue [47]. Paul et al. reported that the porous surface of treated implants and reduced hydrogen gas generation enabled new bone formation around the implant due to increased corrosion resistance [52]. Side effects due to high magnesium levels are rare, as the kidney can regulate the concentration of magnesium ions (Mg^2+^) in the body [53]. Studies show that magnesium ions impregnated in implants can increase the activity and attachment of osteoblasts, promoting new bone formation [54]. The results of this study indicated that magnesium ions generated from the reaction with body fluids could activate cells and influence bone formation. The bone density is improved by increasing the ratio of the contact area between the bone and the implant through anodization surface treatment. It is considered that the supporting ability of the implant is improved due to the increased bone density.

According to micro-CT analysis, a higher bone surface area to bone volume ratio implies a complex and thin structure, while a lower value represents a simple and thick structure [55]. It is a numerical parameter that determines whether the bones are more thinly spread or more thickly gathered. Thus, a lower bone surface area to bone volume ratio denotes thicker bone structure and improved load-bearing capacity [56]. In this study, the ratio of bone surface area to bone volume decreased with calcium addition. Furthermore, the numerical value for the anodization surface treatment group was significantly lower than that for the untreated group, suggesting that anodization surface treatment influenced bone thickness. In particular, the value decreased in the sixth week after degradation compared to the second week in the early stage of degradation. This finding suggested that anodic oxidation surface treatment would have a more positive effect in supporting the bone by thickening the structure during the bone remodeling period when the shape of the bone changes again.

Histological analysis using Goldner’s trichrome staining showed that pure magnesium implants showed the most degradation, and dissolution around the implants was also observed. The anodized group showed more new bone and osteocytes than the untreated group in this study. In histomorphometric analysis, the amount of absorbed magnesium implants was expressed as decreased implant volume in the Mg-Ca systems. A histological analysis by Berglund et al. showed that hydrogen gas production in the early stage of implant degradation was related to decreased implant volume [47]. This study found that magnesium implants rapidly degraded in the first 2 weeks. According to Berglund et al., faster degradation of the alloy in the early stage was attributed to tissue damage or the amount of accumulated body fluid [47]. Moreover, this study confirmed that the amount of remaining magnesium implants increased with the addition of calcium, and the number of absorbed magnesium implants was significantly lower in the anodized surface treatment group. Thus, alloying with calcium and anodization surface treatment creates an oxide layer, which acts as a barrier to the degradation of magnesium, improving its mechanical strength and corrosion resistance [33,57].

Based on histomorphometric analysis, the length of the bone-implant contact area showed a better bone contact rate at 6 weeks than at 2 weeks. The length significantly increased as the amount of calcium added increased, showing the highest value in the 5CMA group. Rahman et al. reported faster bone growth and excellent mechanical bonding in implants with irregular surfaces [58]. Martinez et al. reported that implants with rough surfaces showed higher bone-implant contact rates than implants with flat surfaces [50]. A study investigating the effect of surface roughness on bone-implant bonding reported that improved surface roughness enhanced bone-implant contact [59]. In summary, increased surface roughness of implants improves osseointegration [60]. Increased surface area due to anodization surface treatment enhanced the interface between the bone and the magnesium implant, thereby improving contact with bone.

Histomorphometric analysis showed that the amount of bone tended to increase as the calcium content increased. Moreover, the value significantly increased according to the anodization surface treatment. A study by Davies showed that increased surface area due to increased roughness facilitated the attachment of fibrous proteins (fibrin), inducing osteoconductivity, which increased bone healing and bone formation [61]. Therefore, surface roughness and the oxide layer formed by anodization surface treatment facilitate the reaction with bone, resulting in new bone formation around the implant.

## 5. Conclusions

Within the limitations of this study, biodegradable Mg-Ca systems treated with anodization have potential clinical applications because calcium addition and formed oxide films can slow the initial degradation rate of the implant and produce a surface morphology that promotes sufficient bone healing in the defect. Since the appropriate amount of Mg-Ca systems, optimal anodization treatment conditions, and the exact degradation mechanism have not been elucidated yet, further studies on the bioabsorbability of magnesium implants and scanning methods (such as deep learning technology) that reduce the metal artifacts (seen in micro-CT) are needed in the future. However, our study results have shown the positive effects of alloying and surface treatment on controlling the initial degradation rate of magnesium implants and inducing osteogenesis. The anodization filming of bioabsorbable magnesium systems is considered an appropriate surface treatment method for treating jaw fractures to provide adequate bone healing time meanwhile degradation.

## Figures and Tables

**Table 1:** Experimental groups used in this study.

Surface Treatment	Group	Material	Composition (wt%)	
			Ca	Mg
No Treatment (N)	MN	Pure Mg	-	100
	1CMN	Mg-1Ca	1	99
	5CMN	Mg-5Ca	5	95
Anodic Oxidation Treatment (A)	MA	Pure Mg	-	100
	1CMA	Mg-1Ca	1	99
	5CMA	Mg-5Ca	5	95

**Table 2:** Anodic oxidation condition for anodization process.

Final voltage	120 V
Coating time	15 min
Room temperature	20–25 °C
pH	7–8
Electrolyte solution	Calcium gluconate (4 g/L)
	Sodium hexametaphosphate (3 g/L)
	Sodium hydroxide (6 g/L)

**Table 3:** Condition of micro-computed tomographic scanning.

Parameters	Pre-Set Values
Filter	A1 0.5 mm
Voltage	50 kV
Current	201 mA
Frame averaging	5
Rotation step	0.7°

**Table 4:** Values of micro-computed tomographic scanning parameters.

Parameter Name	Symbol	Unit
Bone mineral density	BMD	g/cm^2^
Bone volume	BV	mm^3^
Bone surface	BS	mm^2^
Bone specific surface	BS/BV	mm^−1^
Trabecular thickness	Tb.Th	mm
Trabecular number	Tb.N	mm^−1^

## Data Availability

All data that support the plots within this paper are available in the main text.

## Abbreviations

The following abbreviations are used in this manuscript:

Mg-Ca	Magnesium–calcium
ROI	Region of interest
Mg	Magnesium
H_2_	Hydrogen gas
Cl^−^	Chlorine ions
Ca	Calcium
Ca^2+^	Calcium ions
MS	Magnesium surface
MV	Magnesium volume
DR	Degradation rate
BS	Bone surface
BV	Bone volume
BS/BV	Bone surface ratio
BMD	Bone mineral density
Tb.Th	Trabecular thickness
Tb.N	Trabecular number
HU	Hounsfield unit
BIC	Bone-to-implant contact ratio
BA	Bone amount
MAO	Micro-arc oxidation
Mg(OH)_2_	Magnesium hydroxide

## References

[1] Song Y., Zhang S., Li J., Zhao C., Zhang X. (2010). Electrodeposition of Ca–P coatings on biodegradable Mg alloy: In vitro biomineralization behavior. Acta Biomater..

[2] Okutan B., Schwarze U.Y., Berger L., Martinez D.C., Herber V., Suljevic O., Plocinski T., Swieszkowski W., Santos S.G., Schindl R. (2023). The combined effect of zinc and calcium on the biodegradation of ultrahigh-purity magnesium implants. Biomater. Adv..

[3] Li Z., Zheng X., Wang Y., Tao T., Wang Z., Yuan L., Han B. (2022). The biomimetics of Mg^2+^-concentration-resolved microenvironment for bone and cartilage repairing materials design. Biomimetics.

[4] Manescu V., Antoniac I., Antoniac A., Laptoiu D., Paltanea G., Ciocoiu R., Nemoianu I.V., Gruionu L.G., Dura H. (2023). Bone regeneration induced by patient-adapted Mg alloy-based scaffolds for bone defects: Present and future perspectives. Biomimetics.

[5] Wang Y., Wei M., Gao J., Hu J., Zhang Y. (2008). Corrosion process of pure magnesium in simulated body fluid. Mater. Lett..

[6] Kannan M.B., Raman R.S. (2008). In vitro degradation and mechanical integrity of calcium-containing magnesium alloys in modified-simulated body fluid. Biomaterials.

[7] Wolf F.I., Cittadini A. (2003). Chemistry and biochemistry of magnesium. Mol. Asp. Med..

[8] Krause A., Von der Höh N., Bormann D., Krause C., Bach F.-W., Windhagen H., Meyer-Lindenberg A. (2010). Degradation behaviour and mechanical properties of magnesium implants in rabbit tibiae. J. Mater. Sci..

[9] Staiger M.P., Pietak A.M., Huadmai J., Dias G. (2006). Magnesium and its alloys as orthopedic biomaterials: A review. Biomaterials.

[10] Yamamoto A., Watanabe A., Sugahara K., Tsubakino H., Fukumoto S. (2001). Improvement of corrosion resistance of magnesium alloys by vapor deposition. Scr. Mater..

[11] Wang H., Shi Z.M., Yang K. (2008). Magnesium and magnesium alloys as degradable metallic biomaterials. Adv. Mater Res..

[12] Reifenrath J., Krause A., Bormann D., Von Rechenberg B., Windhagen H., Meyer-Lindenberg A. (2010). Profound differences in the in-vivo-degradation and biocompatibility of two very similar rare-earth containing Mg-alloys in a rabbit model. Mater. Und Werkst..

[13] Zhang B., Geng L., Huang L., Zhang X., Dong C. (2010). Enhanced mechanical properties in fine-grained Mg–1.0 Zn–0.5 Ca alloys prepared by extrusion at different temperatures. Scr. Mater..

[14] Zhang B., Wang Y., Geng L. (2011). Research on Mg-Zn-Ca Alloy as Degradable Biomaterial, Biomater—Physics and Chemistry.

[15] Bakhsheshi-Rad H., Idris M., Abdul-Kadir M., Ourdjini A., Medraj M., Daroonparvar M., Hamzah E. (2014). Mechanical and bio-corrosion properties of quaternary Mg–Ca–Mn–Zn alloys compared with binary Mg–Ca alloys. Mater. Des..

[16] Ai Y., Luo C., Liu J. (2007). Twinning of CaMgSi phase in a cast Mg–1.0 Ca–0.5 Si–0.3 Zr alloy. Acta Mater..

[17] Son W.w., Zhu X., Shin H.i., Ong J.L., Kim K.h. (2003). In vivo histological response to anodized and anodized/hydrothermally treated titanium implants. J. Biomed. Mater. Res. B Appl. Biomater..

[18] Liu Y., Rath B., Tingart M., Eschweiler J. (2020). Role of implants surface modification in osseointegration: A systematic review. J. Biomed. Mater. Res. Part A.

[19] Makkar P., Sarkar S.K., Padalhin A.R., Moon B.-G., Lee Y.S., Lee B.T. (2018). In vitro and in vivo assessment of biomedical Mg–Ca alloys for bone implant applications. J. Appl. Biomater. Funct. Mater..

[20] Kim J., Lee J., Lee K., Park S., Lim H., Park C., Bae J., Yun K. (2016). Biological evaluation of anodized biodegradable magnesium-calcium alloys. Acta Phys. Pol. A.

[21] Li H., Lu S., Qin W., Han L., Wu X. (2015). Improving the wear properties of AZ31 magnesium alloy under vacuum low-temperature condition by plasma electrolytic oxidation coating. Acta Astronaut..

[22] Sul Y.-T., Johansson C.B., Albrektsson T. (2002). Oxidized titanium screws coated with calcium ions and their performance in rabbit bone. Int. J. Oral Maxillofac. Implant.

[23] Kuhlmann J., Bartsch I., Willbold E., Schuchardt S., Holz O., Hort N., Höche D., Heineman W.R., Witte F. (2013). Fast escape of hydrogen from gas cavities around corroding magnesium implants. Acta Biomater..

[24] Huang J., Song G.-L., Atrens A., Dargusch M. (2020). What activates the Mg surface—A comparison of Mg dissolution mechanisms. J. Mater. Sci. Technol..

[25] Arnett T.R. (2007). Acid–base regulation of bone metabolism. Int. Congr. Ser..

[26] Janning C., Willbold E., Vogt C., Nellesen J., Meyer-Lindenberg A., Windhagen H., Thorey F., Witte F. (2010). Magnesium hydroxide temporarily enhancing osteoblast activity and decreasing the osteoclast number in peri-implant bone remodelling. Acta Biomater..

[27] Xue D., Yun Y., Tan Z., Dong Z., Schulz M.J. (2012). In vivo and in vitro degradation behavior of magnesium alloys as biomaterials. J. Mater. Sci. Technol..

[28] Witte F., Hort N., Vogt C., Cohen S., Kainer K.U., Willumeit R., Feyerabend F. (2008). Degradable biomaterials based on magnesium corrosion. Curr. Opin. Solid State Mater Sci..

[29] Berglund I.S., Brar H.S., Dolgova N., Acharya A.P., Keselowsky B.G., Sarntinoranont M., Manuel M.V. (2012). Synthesis and characterization of Mg-Ca-Sr alloys for biodegradable orthopedic implant applications. J. Biomed. Mater. Res. Part B.

[30] Chen S., Guan S., Li W., Wang H., Chen J., Wang Y., Wang H. (2012). In vivo degradation and bone response of a composite coating on Mg–Zn–Ca alloy prepared by microarc oxidation and electrochemical deposition. J. Biomed. Mater. Res. Part B.

[31] Wang Y., Zheng M., Wu K. (2005). Microarc oxidation coating formed on SiCw/AZ91 magnesium matrix composite and its corrosion resistance. Mater. Lett..

[32] Song G.-L., Atrens A. (2023). Recently deepened insights regarding Mg corrosion and advanced engineering applications of Mg alloys. J. Magnes. Alloy.

[33] Gu X., Li N., Zhou W., Zheng Y., Zhao X., Cai Q., Ruan L. (2011). Corrosion resistance and surface biocompatibility of a microarc oxidation coating on a Mg–Ca alloy. Acta Biomater..

[34] Fischerauer S., Kraus T., Wu X., Tangl S., Sorantin E., Hänzi A., Löffler J.F., Uggowitzer P.J., Weinberg A. (2013). In vivo degradation performance of micro-arc-oxidized magnesium implants: A micro-CT study in rats. Acta Biomater..

[35] Song G.-L., Shi Z. (2013). Anodization and corrosion of magnesium (Mg) alloys. Corrosion Prevention of Magnesium Alloys.

[36] Moreno J., Merlo J.L., Renno A.C., Canizo J., Buchelly F.J., Pastore J.I., Katunar M.R., Cere S. (2023). In vitro characterization of anodized magnesium alloy as a potential biodegradable material for biomedical applications. Electrochim. Acta.

[37] Brar H.S., Ball J.P., Berglund I.S., Allen J.B., Manuel M.V. (2013). A study of a biodegradable Mg–3Sc–3Y alloy and the effect of self-passivation on the in vitro degradation. Acta Biomater..

[38] Witte F., Ulrich H., Palm C., Willbold E. (2007). Biodegradable magnesium scaffolds: Part II: Peri-implant bone remodeling. J. Biomed. Mater. Res. Part A.

[39] Witte F., Kaese V., Haferkamp H., Switzer E., Meyer-Lindenberg A., Wirth C.J., Windhagen H. (2005). In vivo corrosion of four magnesium alloys and the associated bone response. Biomaterials.

[40] Stadlinger B., Lode A.T., Eckelt U., Range U., Schlottig F., Hefti T., Mai R. (2009). Surface-conditioned dental implants: An animal study on bone formation. J. Clin. Periodontol..

[41] Le Guéhennec L., Soueidan A., Layrolle P., Amouriq Y. (2007). Surface treatments of titanium dental implants for rapid osseointegration. Dent. Mater..

[42] Scotchford C.A., Gilmore C.P., Cooper E., Leggett G.J., Downes S. (2002). Protein adsorption and human osteoblast-like cell attachment and growth on alkylthiol on gold self-assembled monolayers. J. Biomed. Mater. Res..

[43] Sezer N., Evis Z., Kayhan S.M., Tahmasebifar A., Koç M. (2018). Review of magnesium-based biomaterials and their applications. J. Magnes. Alloys.

[44] Martinez D.C., Borkam-Schuster A., Helmholz H., Dobkowska A., Luthringer-Feyerabend B., Płociński T., Willumeit-Römer R., Święszkowski W. (2024). Bone cells influence the degradation interface of pure Mg and WE43 materials: Insights from multimodal in vitro analysis. Acta Biomater..

[45] Prakash T. (2012). Review on nanostructured semiconductors for dye sensitized solar cells. Electron. Mater. Lett..

[46] Martinez H., Davarpanah M., Missika P., Celletti R., Lazzara R. (2001). Optimal implant stabilization in low density bone. Clin. Oral Implant. Res..

[47] Berglund I.S., Jacobs B.Y., Allen K.D., Kim S.E., Pozzi A., Allen J.B., Manuel M.V. (2016). Peri-implant tissue response and biodegradation performance of a Mg–1.0 Ca–0.5 Sr alloy in rat tibia. Mater. Sci. Eng. C Mater. Biol. Appl..

[48] Tao M., Cui Y., Sun S., Zhang Y., Ge J., Yin W., Li P., Wang Y. (2025). Versatile application of magnesium-related bone implants in the treatment of bone defects. Mater. Today Bio.

[49] Hung C.-C., Chaya A., Liu K., Verdelis K., Sfeir C. (2019). The role of magnesium ions in bone regeneration involves the canonical Wnt signaling pathway. Acta Biomater..

[50] Ali A., Ikram F., Iqbal F., Fatima H., Mehmood A., Kolawole M.Y., Chaudhry A.A., Siddiqi S.A., Rehman I.U. (2021). Improving the in vitro degradation, mechanical and biological properties of AZ91-3Ca Mg alloy via hydrothermal calcium phosphate coatings. Front. Mater..

[51] Mohamed A., El-Aziz A.M., Breitinger H.-G. (2019). Study of the degradation behavior and the biocompatibility of Mg–0.8 Ca alloy for orthopedic implant applications. J. Magnes. Alloys.

[52] Paul W., Sharma C.P. (2006). Nanoceramic matrices: Biomedical applications. Am. J. Biochem. Biotechnol..

[53] Vormann J. (2003). Magnesium: Nutrition and metabolism. Mol. Asp. Med..

[54] Zreiqat H., Howlett C., Zannettino A., Evans P., Schulze-Tanzil G., Knabe C., Shakibaei M. (2002). Mechanisms of magnesium-stimulated adhesion of osteoblastic cells to commonly used orthopaedic implants. J. Biomed. Mater. Res..

[55] Ulrich D., Van Rietbergen B., Laib A., Ruegsegger P. (1999). The ability of three-dimensional structural indices to reflect mechanical aspects of trabecular bone. Bone.

[56] Parkinson I.H., Forbes D., Sutton-Smith P., Fazzalari N.L. (2010). Model-Independent 3D Descriptors of Vertebral Cancellous Bone Architecture. J. Osteoporos..

[57] Blawert C., Dietzel W., Ghali E., Song G. (2006). Anodizing treatments for magnesium alloys and their effect on corrosion resistance in various environments. Adv. Eng. Mater..

[58] Rahman M., Dutta N.K., Roy Choudhury N. (2020). Magnesium alloys with tunable interfaces as bone implant materials. Front. Bioeng. Biotechnol..

[59] Shalabi M., Gortemaker A., Hof M.V.t., Jansen J., Creugers N. (2006). Implant surface roughness and bone healing: A systematic review. J. Dent. Res..

[60] Uppal G., Thakur A., Chauhan A., Bala S. (2014). Magnesium based implants for functional bone tissue regeneration—A review. J. Magnes. Alloy.

[61] Davies J. (1998). Mechanisms of endosseous integration. Int. J. Prosthodont..

